# Metabolomics for Crop Breeding: General Considerations

**DOI:** 10.3390/genes12101602

**Published:** 2021-10-12

**Authors:** Dmitry Y. Litvinov, Gennady I. Karlov, Mikhail G. Divashuk

**Affiliations:** All-Russia Research Institute of Agricultural Biotechnology, Timiryazevskaya Street, 42, 127550 Moscow, Russia; karlovg@gmail.com (G.I.K.); divashuk@gmail.com (M.G.D.)

**Keywords:** metabolites, metabolic pathways, lipids, mass spectrometry, nuclear magnetic resonance, sample preparation, marker-assisted selection, mQTL, mGWAS, genetically modified crops

## Abstract

The development of new, more productive varieties of agricultural crops is becoming an increasingly difficult task. Modern approaches for the identification of beneficial alleles and their use in elite cultivars, such as quantitative trait loci (QTL) mapping and marker-assisted selection (MAS), are effective but insufficient for keeping pace with the improvement of wheat or other crops. Metabolomics is a powerful but underutilized approach that can assist crop breeding. In this review, basic methodological information is summarized, and the current strategies of applications of metabolomics related to crop breeding are explored using recent examples. We briefly describe classes of plant metabolites, cellular localization of metabolic pathways, and the strengths and weaknesses of the main metabolomics technique. Among the commercialized genetically modified crops, about 50 with altered metabolic enzyme activities have been identified in the International Service for the Acquisition of Agri-biotech Applications (ISAAA) database. These plants are reviewed as encouraging examples of the application of knowledge of biochemical pathways. Based on the recent examples of metabolomic studies, we discuss the performance of metabolic markers, the integration of metabolic and genomic data in metabolic QTLs (mQTLs) and metabolic genome-wide association studies (mGWAS). The elucidation of metabolic pathways and involved genes will help in crop breeding and the introgression of alleles of wild relatives in a more targeted manner.

## 1. Introduction

**Figure 1:**
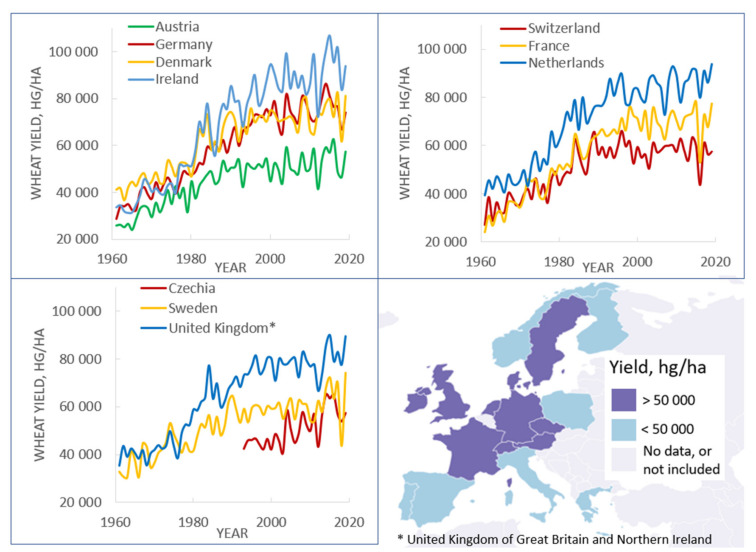
Wheat yield in 1961–2019 in European countries with a current yield of over 50,000 hg/ha. Countries are divided into three panels to avoid overlapping graphs. (Source: FAOSTAT, online database, http://www.fao.org/faostat/en/#data/QCL, accessed on 19 September 2021. Map was drawn at http://mapinseconds.com, accessed on 20 September 2021).

The rapid accumulation of genomic data, the development of genetic approaches and their application in plant breeding over the past decades have significantly modernized plant breeding. Approaches based on the association of DNA markers with beneficial traits, such as marker-assisted selection (MAS), have become a common tool in plant breeding [1,2,3,4]. Quantitative trait loci (QTL) mapping and genome-wide association studies (GWAS) are currently widely applied for the identification of genes or genomic regions associated with traits of interest [5,6,7]. Despite this progress, producing new crop varieties does not always result in noticeable success [8,9]. For example, trends in wheat yields in France, Germany, Great Britain and other European countries with high modern yields (over 50,000 hg/ha) have gradually reached a plateau over the past decade (Figure 1).

This stagnation in wheat yields occurs at a time when a large number of loci are identified that are responsible for many of the beneficial characteristics of wheat (collected in WheatQTLdb [10]).

To further improve yields, a better understanding of the physiological processes that underlie the beneficial properties of crops is needed. Other “omics” besides genomics, such as transcriptomics, proteomics and metabolomics, provide valuable information about the functions of the uncharacterized genes and molecular mechanisms that are involved in specific plant processes.

Metabolomics studies metabolite profile, a repertoire of small molecules of a cell, tissue or biological fluid and differences in profiles, depending on environment and genotypes [11].

The level (concentration) of a specific metabolite depends on the activity of metabolic (biochemical) pathways that results in its generation and its utilization, as well as the pathways that consume or generate the precursors of the given metabolite. Many proteins are involved in these processes directly as enzymes, and indirectly, as regulatory proteins, proteins of intracellular transport and scaffold, proteins that maintain the optimal redox state, etc. Further, the expression and performance of these proteins depend on the temperature, availability of nutrients and stresses. Thus, the level of metabolites integrates multiple gene × gene (G×G) and gene × environment (G×E) interactions and “serves as direct signature of biochemical activity” [11,12].

Transcriptomics and proteomics, while valuable tools, still overlook much of the interaction between G×G and G×E. Moreover, the allelic forms of a gene can barely be differentiated using transcriptomics and can hardly be distinguished using proteomics. Metabolomics would differentiate these allelic forms if they were functionally different and affect the level of metabolites. Indeed metabolomics and its integration with other “omics” is a powerful approach for the elicitation of meaningful information of plant biochemical and physiological mechanisms and improving crops. By now, the most fruitful results are derived in metabolomics on plant stress response [12].

In this review, we (1) argue the limitations of purely genetic tools for the plant traits improvement and the growing need for functional studies. We then focus on (2) metabolites as the basal elements of metabolomics and metabolism as the central organizational framework of metabolomics, (3) metabolomics as a set of experimental methods with their strengths and weaknesses and (4) we will illustrate the different types of metabolomics research with examples related to crop breeding.

## 2. The Growing Need in Functional Knowledge for Crop Improvement

In the last decades, breeders, using direct genetics approaches, have significantly improved yields and other desirable crop traits. A recent genetic analysis and QTL mapping of dozens of traits using 16 historical UK bread wheat varieties and over 500 recombinant inbred lines (RILs) derived from them revealed the genetic basis of the improvement of wheat crop from 1935 to 2004. This improvement appeared to be based on (1) the search for the optimal combination of alleles with little effect by “shuffling” these alleles for poly-genic traits and (2) the introduction of a small number of loci with a large effect from wide crossings, such as *Rht* semi-dwarfing alleles and introgression of large disease-resistant genomic segments from other species. This study indicated that most QTL are likely to have pleiotropic effects that make it harder to improve crops, as improving one trait can negatively affect others; for example, an increase in yield usually results in a decrease in protein content [13].

Simulation models showed the inefficiency of blind-breeding for the generation of novel beneficial combinations of alleles. However, it was concluded that the selection for dozens of loci makes crop improvement possible. It is expected that further improvement of crops will be assisted by genomic selection and driven by the introgression of selected exotic alleles and genome-wide introduction of diverse haplotypes [13].

Indeed, harnessing the genetic potential of wild relatives is seen as a powerful solution for further crop improvement. The wild relatives are much more genetically diverse and better fit to environmental stresses than domesticated cultivars and can be sources of new alleles, especially for disease resistance [14]. Furthermore, it was reported that roughly 56,000 domesticated hexaploids, 19,000 domesticated tetraploid and almost 4000 crop wild relatives of wheat, in the collections of The International Maize and Wheat Improvement Center’s (CIMMYT) and International Center for Agricultural Research in the Dry Areas (ICARDA) germplasm banks, still have unexplored genetic diversity [15].

Nevertheless, breeding new varieties of crops with improved traits inevitably becomes more and more difficult as the best existing alleles of genes are identified and maintained in elite cultivars. The majority of agronomic traits are complex traits that are determined by several or many genes with varying degrees of influence (effect) on the trait [16]. It can be assumed that for the crop improvement, genes with a strong effect are optimized first, then breeders optimize the combination of more genes with a weaker effect, and gradually, to continue improving the plant, a very large number of genes need to be optimized.

The situation becomes even more complicated if considering the pleiotropic effect of genes and epistasis, that is, the interaction between genes. The epistasis manifests as enhancing (positive epistasis) or inhibiting (negative epistasis) the effect of a gene on a trait by other genes. It also means that the same allele of a gene can contribute to a trait differently in a different genetic background [17,18]. Epistasis was suggested as an explanation of missing heritability [19]. The idea of epistasis was extended in the omnigenic theory, which states that complex traits are controlled by all genes of the organism [20].

The determination of the full effect of epistasis is extremely difficult from experimental and computational points of view [21]. QTL mapping and GWAS do not take into account epistasis and consider all genetic variations as independent factors influencing the trait and calculate the cumulative effect of variants as additive. In some modifications, these genetic mapping methods apply very limited models for defining epistasis, but even with them, it is possible to notice the presence of epistasis [16]. Some efforts are being made to reveal epistatic effects in plant and human genomes. However, the search for gene–gene interaction requires at least 10–100-fold bigger sample sizes than are required for equivalent additive gene effects determined in QTL and GWA studies [22]. Currently, such genetic–trait association studies are performed using hundreds or thousands of lines at a time, such as 4500 bread wheat lines in a study on wheat genetic diversity [23]. Increasing the number of lines by a factor of 10–100 would be a difficult task. In another work, the number of samples for determining the interaction of only two genes using GWAS is estimated at 10,000 while there are millions of millions of gene pairs [20]. It can be summarized that currently, we do not have adequate tools to comprehensively evaluate the epistasis [21].

As with plants, the association of human traits with genotypes using SNPs arrays is a powerful tool. In humans, it allowed the revealing of “thousands of genetic variants affecting hundreds of human traits” [24]. Nonetheless, in 2020, the National Human Genome Research Institute, the leading institution of Human Genome Project, in the Strategic vision for improving human health at The Forefront of Genomics, stated that the connection of specific genomic variants to phenotype, beside the cases of monogenic traits and some more complex cases, is challenging, and the progress in this area requires global collaboration and the integration of different experimental approaches beyond the genomics [25]. The ultimate goal is to identify the molecular mechanisms responsible for the appearance and manifestation of the traits of interest. In a closer perspective, other “omics” can determine non-genomic markers associated with desired traits, such as grain yield. Aside from the major “omics”—transcriptomics, proteomics, and metabolomics with lipidomics—there are more specialized branches, such as epigenomics, miRNAomics, interactomics [26], phospho-proteomics (global analysis of protein phosphorylation status) [27] and kinomics (activity of the complete set of kinases) [28], that can generate a wealth of information. If “phenotype” refers to the observable physical properties of an organism [29,30], then each of the “omics”, besides genomics, describes the phenotype of an organism in its own manner. The further the “omics” from the genome on the way of the translation of genetic information to phenotype, the more it reflects the complex interactions between genes and between the genes and the environment. Metabolomics, which characterizes the profiles of small molecules, is the last in the chain of major “omics”—it follows after transcriptomics and proteomics. Thus, metabolomics reflects the largest contribution of the gene–gene and gene–environment interactions of all “omics”, and in the chain of the deployment of the genetic program into the organism stands just before the final morphological phenotype [11,31]. Sometimes the metabolome, proteome or transcriptome is called intermediate endophenotypes [32] or intermediate phenotype [33,34]. However, the term “intermediate phenotype” has other meanings, such as a phenotype between the two extreme phenotypes of the parents, and therefore, this term sounds ambiguous.

In some cases, a metabolite can be an essential desirable or undesirable trait, such as specific compounds in medicinal plants [35], sugar in sugar cane and sugar beet, organic acids, vitamins, flavonoids, flavors in fruits [36] or unwanted anti-nutritional factors, such as saponins, phytic acid [37]. In other cases, a metabolite can be a precursor for an essential trait, such as sugars and amino acids, as building blocks for storage polysaccharides and proteins in cereals [38]. Universally, metabolites can reflect the environmental conditions and the adaptation to environmental stresses.

As an ultimate goal, the metabolome of a plant should be deciphered as a set of biochemical pathways with known enzymes and transporters and with measured fluxes of the key metabolites along different biochemical pathways and between the compartments [38,39,40]. This knowledge should allow the selection of optimal alleles not by random testing of different alleles but by using an educated choice. It also should allow the introduction of the specific mutations to create alleles beyond naturally available variants and, if the social acceptance of genetically modified plants will prevail, rationally design the optimal biochemical makeup of the plants [41].

## 3. Metabolism and Metabolites

Metabolism, as a set of specific chemicals and, in the case of photosynthesis, photochemical processes, is one of the central attributes of life. Metabolites are small molecules, usually considered in the range of molecular weight of up to 1500–2000 Da or g/mol, that are formed during metabolism [42]. Virtually all plants generate metabolites by assimilating carbon dioxide as the sole carbon source. A few metabolites are non-carbon molecules, such as hydrogen peroxide, ammonium derived from assimilated nitrates and pyrophosphate formed during ATP hydrolysis. All metabolites are divided into two groups—primary (central) metabolites and secondary (specialized) metabolites.

Primary (central) metabolites are small molecules that are absolutely required for the viability of a cell in an organism. They participate in the central carbon metabolism, metabolism of amino acids, nucleotides and lipids and other processes that have a lot in common with different branches of living organisms [43]. The number of primary metabolites in plants is estimated under 10,000 and is assumed slightly higher than in other biological kingdoms since plants are autotrophs and require photosynthetic metabolic pathways [44]. The perturbation of these metabolites is of interest since their availability affects the ability of the cell to generate energy, build the necessary macromolecules, cellular structures and generate secondary metabolites. Among the primary metabolites, several small molecules stand out due to their high thermodynamic instability, which allows them to participate in and promote catabolic reactions. These molecules are ATP, which readily donates phosphoryl or nucleotidyl groups and provides energy in reactions coupled with its cleavage; electron donor nicotinamide adenine dinucleotide (NADH) and its phosphorylated form NADPH, acyl group donor acetyl coenzyme A (acetyl-CoA) and molecules that are donors of several other groups: S-adenosylmethionine (SAM), isopentenyl pyrophosphate (IPP), ADP-glucose (adenosine diphosphate glucose) and UDP-glucose (uridine diphosphate glucose) [45].

Secondary (specialized) metabolites are small molecules that are not necessary for the viability of individual plant cells but are essential for the viability of the organism. Secondary metabolites mediate intercellular regulation and communication. Plants are sessile in nature, and chemicals play a major role in the plant–environment interaction. These secondary metabolites protect plants against biotic and abiotic stresses, protect from herbivores, attract pollinating and seed-dispersing insects and animals. The generation of a number of secondary metabolites are responses to environmental stress [46].

Given the functional and regulatory role of small molecules, the study of the metabolome can be expected to identify small molecules that are associated with important agronomic traits.

The total number of metabolites produced by the plant kingdom is estimated at approximately 200,000—1 mln, and a single plant species can generate dozens of thousands of metabolites [44,47]. The recent efforts to compile the known natural products from all currently existing databases resulted in the collection of over 400,000 non-redundant substances with molecular masses below 4000 Da. For 32% of these substances, there is an indication in which organisms they are contained, and of these substances, 88,000 are of plant origin, which is 66% of all natural products with an indication of origin [48,49].

Different cell organelles are responsible for different metabolic pathways. Chloroplasts are the sites of abundant generation of energy (ATP) and reducing potential (NADPH) equivalents in light-dependent reactions of photosynthesis. In following light-independent reactions of photosynthesis, known as Calvin or reductive pentose phosphate (RPP) cycle, the molecules of ATP and NADPH are consumed for the incorporation of CO_2_ to ribulose-1,5-bisphosphate (RuBP) followed by the hydrolysis of the resulted product into 3-phosphoglycerate (3PG), its reduction to glyceraldehyde-3-phosphate (G3P) and resynthesizing of RuBP. The major gain from the Calvin cycle is G3P, which is generated in excess of what is needed for the continued functioning of the Calvin cycle and is used for carbohydrate synthesis in the cytoplasm. Other metabolic pathways in chloroplasts are fatty acid synthesis and the shikimate pathway that links primary and secondary metabolisms by producing chorismite, the common precursor for aromatic amino acids and a range of secondary metabolites [50]. Some secondary metabolites are also stored in plastids.

Mitochondria are the site of oxidative phosphorylation that generates ATP and the Krebs cycle that generates ATP and NADPH, the important metabolites that provide reducing potential. Mitochondria also harbor several central metabolism pathways, such as the citrate cycle and the metabolism of amino acids. The study of mitochondria metabolites is still challenging. A Mito-AP protocol for plant mitochondria isolation from the leaves of genetically modified *Arabidopsis* has been recently developed. The transgenic plant expresses a mitochondrial outer membrane protein fused to a GFP: Strep-tag that allows a quick (0.5–1 h) isolation of mitochondria using affinity beads. Isolated mitochondria maintain membrane integrity, membrane potential and respiratory activity. However, the ratios of NADH/NAD and ATP/ADP are not normal for active mitochondria, which indicates the inactivation of mitochondria during the isolation. Thus, even this quick and gentle method is suitable for the analysis of the entire group of metabolites, such as ATP + ADP + AMP, and for stable metabolites, such as lipids [51].

Cytosol harbors several important metabolic pathways: glycolysis, the oxidative pentose phosphate pathway, amino acid biosynthesis, fatty acid metabolism, one-carbon metabolism (methionine and folate cycles) and vitamin B5 synthesis [52]. Vacuoles of plant cells are mainly storage organelles. They store inorganic nitrates and phosphates, sugars and amino acids. Amino acids are released in vacuoles as a result of the degradation of proteins since plants do not have lysosomes, and the catabolism of proteins occurs in vacuoles. Furthermore, vacuoles accumulate and store secondary metabolites [53].

In many cases, the synthesis of a metabolite requires multiple enzymes localized in different compartments. For example, the synthesis of the plant hormone jasmonic acid begins in the chloroplast and ends in the peroxisome [54].

The highest concentration of some metabolites can be in sites different from the synthesis. At the cellular level, such storage organelle is often vacuole. At the level of the whole plant, there are sink organs (usually seeds and roots), where sugars and amino acids are transported from the source organ (leaves) and stored in a polymeric form.

## 4. Lipids—A Class of Metabolites with Distinct Properties. Classification and Metabolism

Lipids are a group of structurally different molecules that share the common property—they are hydrophobic (lipophilic) or amphipathic (contains both hydrophobic and hydrophilic parts), due to which they form bilayer membranes, lipid droplets and bind with the hydrophobic residues of proteins [55]. Since lipids differ from other metabolites in their functions in the cell and in the experimental methods used to study them, lipidomics, although it can be considered as a subdivision of metabolomics, stands apart.

Lipids are necessary for any cell to form a plasma membrane and membranes of cellular organelles and vesicles. Aside from the creation of compartments, membranes serve as scaffolds for integral membrane proteins and proteins associated with membrane surfaces. The organization of biological membranes were studied mostly for human and animal cells and revealed that the lipid composition varies between cell organelles and plasma membrane. Further, the membranes are not uniform laterally (in the plane of membrane) and have some microdomains enriched with specific lipids, such as lipid rafts enriched by cholesterol and sphingomyelin [56,57]. Even more, two leaflets, at least for plasma membrane, are different in their lipid composition, despite the energy expenses that are required to maintain this difference.

Aside from the structural role, lipids also serve as storage for energy that can be released through oxidation and for carbon building blocks for transformation into other metabolites, including some vitamins (A, D, E, K), coenzyme Q, carotenoids and signal molecules, such as a phytohormones brassinosteroids and jasmonates.

The chemical diversity of lipids is high. The LipidBank database contains over 7000 natural lipid structures, including fatty acids, glycerolipids, sphingolipids, steroids and vitamins (http://www.lipidbank.jp/ accessed on 1 October 2021; [58]). Lipid Metabolites and Pathways Strategy (LIPID MAPS) lists in their lipid database LMSD, 24,285 biologically-relevant manually-curated lipid structures (the total number of structures including computer-generated is over 46,000 entries) (https://www.lipidmaps.org/resources/databases/ accessed on 1 October 2021; [59]). Moreover, LIPID MAPS maintains the LMPD database of lipid-related genes and proteins that currently contains 1829 genes and 2447 proteins for *Arabidopsis thaliana* (https://www.lipidmaps.org/resources/databases/index.php?tab=lmpd accessed on 1 October 2021; [60]).

The majority of the lipidomic research is focused on humans and animals; thus, the majority of the entries are not plant lipids. However, the plant lipidomics are being developed. The classification of naturally occurring lipids developed by LIPID MAPS divides all biological-relevant lipids into eight categories based on their chemical structure. The most numerous is the fatty acyls category, with 8219 curated structures (cs), glycerolipids (285 cs) with fats and oils in this category, glycerophospholipids (1645 cs) the major constituents of the membranes belongs to this category, sphingolipids (1707 cs), sterol lipids (3056 cs), prenol lipids (1536 cs), sacccharolipids (32 cs) and polyketides (6996 cs). The fatty acyls category with 8219 curated structures in the LIPID MAPS database is the most numerous category of lipids. Fatty acids that are the members of this category are usually present in plants and other organisms in their free form in very low concentrations. However, in chemically-bound forms, fatty acids constitute a significant part of any plant tissue or cell lipid mass, being the fundamental building blocks of more complex lipids, such as phospholipids and triglycerides. The fatty acyls category also includes oxylipins [61], such as phytohormones jasmonic acids, and products of fatty acids enzymatic oxidation and oxidation by reactive oxygen species, such as hydroperoxy fatty acids, phytoprostanes and phytofurans.

Lipid droplets (also known as lipid or oil bodies, oleosomes and spherosomes) originate from the endoplasmic reticulum and are the storage organelles for triacylglycerols for energy and for carbon backbone [62,63].

Lipids are relatively big molecules compared to the majority of other metabolites with a relatively complex structure. The utilization of generally accepted universal guidelines defined by the International Union of Pure and Applied Chemists and the International Union of Biochemistry and Molecular Biology (IUPAC-IUBMB) Commission on Biochemical Nomenclature (https://iupac.qmul.ac.uk/lipid/, accessed on 1 October 2021) for lipids results in bulky systematic names. Moreover, experimental techniques, despite the constant development, still often reveal just partial structural information [64] that makes a problem for the application of systematic names. A shorthand nomenclature was developed to standardize and simplify reporting lipid species, which is also useful for experimental findings since it is suitable for reporting lipids molecules at the different levels of revealing their exact structure [65].

For example, a glycerolipid diacylglycerol at different levels of resolution would be recorded as DG 34:1 (Species Level; 34 stands for total carbon atoms in acyl chains, 1 is the number of double bonds in these chains), DG 16:0_18:1 (Molecular Species Level; the acyl chains are now resolved as fully saturated 16-carbon and 18-carbon chain with one double bond), DG 16:0/18:1/0:0 (*sn*-Position Level; now the hydroxyl groups in glycerol that are esterified by acyl chains are defined), DG 16:0/18:1(9Z)/0:0 (Full Structure Level; 9Z means the position of a double bond between the 9th and 10th carbon atoms counting from acyl carbon and cis geometry of the double bond).

Lipid synthesis in plants occurs in plastids (chloroplasts), cytoplasm (in the endoplasmic reticulum, ER) and to some extent in mitochondria. Fatty acids (FA) are an essential part of most lipid molecules that provide hydrophobic properties and enable them to generate lipid bilayer, or lipid monolayer and hydrophobic core in lipid droplets.

The essential role for the synthesis of FA plays plastids, where palmitic (16:0) and stearic (18:0) acids are synthesized by an FA synthase complex of seven polypeptides encoded by nuclear DNA [66], using a substantial number of NADPH molecules (14 for palmitic acid). The plant FA synthesis site is different from the animal cells and other eukaryotes, where FA synthesis happens in ER in the cytoplasm. Further, in plastids, the stearic (18:0) acid is converted to monounsaturated oleic (18:1) acid. The synthesis of various glycerolipids from FA occurs both in plastids and in ER in the cytoplasm [67].

To release the energy stored in triacylglycerols (TAG) in lipid droplets, cells first carry out lipolysis by TAG lipases located on the surface of peroxisomes. Then FA catabolism occurs by β-oxidation, which is the major pathway for the utilization of FAs for the generation of energy and for building carbon skeletons for other metabolites. This process of fuel germination and early seedling growth provides energy in leaves in the form of ATP for stomatal opening after the plant transfer from dark to light [68]. Unlike mammals, where β-oxidation occurs in mitochondria, in plants, β-oxidation occurs in peroxisomes. The FAs that are released during lipolysis are transported into peroxisomes by ATP binding cassette (ABC) transporter subfamily D of the peroxisomal membrane. β-Oxidation results in the degradation of FA to acetyl-CoA.

Thus, besides the membranes and lipid droplets, the lipid-related organelles in plants are plastids, peroxisomes and in a lesser degree in mitochondria.

## 5. Genetically Modified Plants with Altered Activity of Metabolic Enzymes

Metabolomics is a younger field than other omics and is just beginning to contribute to crop improvement. Beyond metabolic markers, metabolomics has to identify biochemical pathways underlying the desired traits. In this, metabolomics is similar to biochemistry but uses another, more data-rich approach. To evaluate the applicability of biochemical knowledge to crop improvement, the commercialized genetically modified (GM) crops with altered metabolic activities were selected from the GM Approval Database that is maintained by the International Service for the Acquisition of Agri-biotech Applications (ISAAA). About 50 metabolic enzymes that are directly involved in metabolism were found altered in this set of GM crops (see Table 1).

With regard to metabolic changes caused by the introduction of additional metabolic enzymes or suppression of the activities of endogenous enzymes, almost 30 types of metabolically altered GM have been found; for some types, many crop accessions have been reported. These alterations were done in fifteen different crops, mainly in staple food crops (four in maize, four in soybeans, two in potato), oilseeds (four in Argentine canola), fruits (two in tomato) and in industrial crops (two in sugarcane).

In most cases (41 times in the considered GM crops) the genetic intervention was aimed to achieve a gain of function. For this, the heterologous genes were transferred from various sources. The most abundant source of transgenes in our set of GM crops was marine microalgae, reflecting the need for many different enzymes to alter the oil/fatty acid properties and good fit of algae enzymes for the generation of desired fatty acid variants. Twelve genes from various microalgae were employed. For the same purpose, three genes from oomycetes and one gene from moss from fungi and yeast were also used.

In other GM crops, two fungal genes have been used to break down plant small-molecule phytate in maize (BVLA430101 from Origin Agritech (China)) and Argentine canola (Phytaseed™ Canola from BASF) to release phosphorus and make it available to humans and monogastric animals.

In some cases, the loss of function was employed (~20 times considering GM crops) that was achieved by RNA-mediated gene silencing. This means the Innate^®^ Cultivate and other GM potato cultivars were generated by J.R. Simplot Co. suppressing Asparagine synthetase 1 (Asn1), polyphenol oxidase 5 (Ppo5), starch phosphorylase L (PhL), glucan water dikinase (R1) and, in some cultivars, vacuolar invertase (VInv). The resulted GM potato does not develop black-spot bruise due to less Ppo5; when fried, it produces much less acrylamide due to less asparagine (lower Asn1 activity) and is not as brown as the original potato due to having fewer reducing sugars (less R1 and VInv activity) [69].

Many GM crops have stacked many modified genes. For example, Argentine canola (*Brassica napus*) LBFLFK (BASF) received 10 genes from microalgae, oomycetes and moss to increase omega-3 long-chain (20 or longer carbons) polyunsaturated fatty acids (PUFA), the health-promoting lipids that are contained in fish oil. Indeed, LBFLFK produces seed oil that contains ~7% eicosapentaenoic acid (EPA; 20:5n-3), one of the major omega-3 PUFA [70].

The introduction of two variants of choline dehydrogenase from different bacteria, EcBetA and RmBetA, by PT Perkebunan Nusantara XI (Persero) into sugarcane improved drought stress tolerance. Choline dehydrogenase produces glycine betaine, a metabolite that has been shown to have a protective effect against abiotic stresses (osmolyte; see Section 10.1). The first cultivar NXI-1T (with choline dehydrogenase EcBetA) received approval in Indonesia in 2011 for food and in 2013 for cultivation, and two other cultivars with choline dehydrogenase RmBetA, NXI-4T and NXI-6T, received approval in 2013 in Indonesia for cultivation, food and feed, and for cultivation and food, respectively (Table 1).

Overall, metabolic alterations in GM suggest that genes from taxonomically very distant biological sources work well in crops.

## 6. Methods of Metabolomics

### 6.1. General Considerations

Unlike genomics and similarly to transcriptomics, proteomics and other “omics”, metabolomic profiles in the organism differ in the spatial aspect (differ between organs, tissues, cell types and subcellular structures), in the temporal aspect (differ at different stages of development, phases of the cell cycle for dividing tissues and diurnal cycle) and depend on environmental conditions (temperature, light, water and nutrient availability, stress factors). Thus, for metabolomic experiments, the consistency in growth conditions of plants and consistency in sample preparation is of great importance. Moreover, some metabolites are not stable chemicals and get hydrolyzed or oxidized quickly, for example, NADPH. Special precautions should be considered for the analysis of such metabolites.

Metabolic studies can be targeted when a set of certain metabolites that are expected to be affected under the experimental conditions is analyzed, or untargeted (global), when a wide range of metabolites is analyzed without any preference for specific metabolites. In terms of determining the chemical structure of metabolites, metabolic studies can result in the list of metabolites with known structures or provide just “molecular features” (or “metabolite features” or “mass features” for mass spectrometry; MF)—a list of peaks, which correspond to undefined metabolites. Each MF, such as the m/z peak obtained by mass spectrometry or chemical shift peak obtained by nuclear magnetic resonance, can originate from different metabolites, and one metabolite can produce several MFs. MFs can be used as markers associated with desired traits of a plant. Metabolic studies that identify MFs are suitable for the classification of samples and are commonly referred to as “metabolic fingerprinting” rather than “metabolic profiling” [71,72,73]. Whether the chemical structures of the metabolites are determined or metabolic study results in MF data, usually the concentrations of metabolites are obtained in relative units. A reliable absolute quantification (i.e., quantification in units, such as mg/mL of the sample) can be done by the addition of the known amount of stable isotope-labeled metabolite of interest to the sample as an internal control.

### 6.2. Mass Spectrometry

Two major analytical technologies are currently used in metabolomics for the identification of small molecules—mass spectrometry (MS) and nuclear magnetic resonance (NMR). MS systems, apart from rare and highly advanced systems, such as Fourier-transform ion cyclotron resonance (FTICR), can only resolve a relatively limited number of small molecules at a time. Therefore, they are coupled with chromatographic systems to separate a complex mixture of metabolites and reduce the number of substances entering the MS at each point in time. The type of chromatography—gas chromatography (GC) or liquid chromatography (LC)—determines the possible ionization methods. Thus, MS coupled with GC (GC-MS) and MS coupled with LC (LC-MS) have specific features beyond the differences in chromatographic separation.

The separation using GC can only be performed for relatively volatile compounds, and these compounds should be thermally stable at the conditions of separation (usually in the range 150–450 °C). To achieve it, metabolites are subjected to chemical derivatization prior to GC-MS analysis for converting of non-volatile compounds to volatile products. Several classes of metabolites can be analyzed by this method: amino acids, various sugars (sugars, sugar phosphates, sugar acids and sugar alcohols) and organic acids [73]. GC-MS is suitable for the determination of phytohormones, such as salicylic acid (SA), jasmonic acid (JA), jasmonoylisoleucine (JA-Ile), indole-3-acetic acid (IAA), in-dole-3-carboxylic acid (ICA), indole-3-butyric acid (IBA) and 2-*cis*,4-*trans*-abscisic (ABA), and phytohormone precursors, such as benzoic acid (BA), trans-cinnamic acid (CA) and 12-oxo-phytodienoic acid (OPDA) [74,75]. However, LC-MS is now more commonly used for the determination of phytohormones than GC-MS [76].

Analysis of lipids, which is often considered to be related to but distinct from the area of metabolomics, called lipidomics, also can be performed by GC-MS, although it is not the preferred method nowadays [77,78]. When using GC-MS, part of the structural information is lost, since triglycerides are hydrolyzed for subsequent chemical derivatization, and in the resulting sample there is no way to determine which fatty acid residues were in which positions in the original phospholipids, triglycerides, and other lipid molecules.

Electron (or electron impact) ionization (EI) is a common ionization method in GC-MS, and besides ionization, it causes the fragmentation of molecules. As a result of EI, each chemical substance generates a specific pattern of fragments, and the comparison of this pattern with patterns in a library allows the identification of the metabolite.

The number of identified metabolites in the sample depends on the type of mass analyzer. The higher the mass resolution (the ability to distinguish two adjacent ions), the ion mass range, and the acquisition rate (the rate of the scan for the full mass range), the more individual metabolites can be identified and quantified in a sample. The most affordable GC-MS systems are equipped with a quadrupole (Q) analyzer and are well suited for targeted metabolomics when high sensitivity is not required, and are also suitable for non-targeted metabolomics to measure up to 100 of the most abundant metabolites (practically speaking, several dozen of metabolites). GC-MS systems with a time of flight (TOF) analyzer can measure up to 1000 metabolites in untargeted metabolomic studies (practically speaking, several hundred metabolites), and can also be used in targeted metabolomics for measurements of low abundance metabolites that are obscured by major metabolites in Q analyzers due to their lower resolution. Orbitrap and especially Fourier transform ion cyclotron resonance (FTICR) are high-end analyzers that are very powerful and can measure roughly the entire metabolome [79].

MS systems with triple quadrupoles (QqQ) allow separation of selected ion(s) for the fragmentation step, thus providing tandem (MS/MS) capability for the analysis. The ions that are generated from the precursor (parent) ion provide additional structural information on the parent ion in the untargeted metabolomic approach. The MS/MS capability is extremely useful in the targeted metabolomics approach for accurate quantification of low-abundant metabolites, such as phytohormones. While several metabolites can produce the ions of the same or near the same mass-to-charge ratios (m/z), the MS/MS systems are capable of picking specific ion(s) for further destruction that produces product (daughter) ions with a different m/z depending on the structure of the original (precursor or parent) ion. Monitoring such specific product ion allows a specific quantitative determination of the concentration of the metabolite of interest. This tandem MS quantification method is called SIM (Single Son Monitoring). Likewise, the MRM (Multiple Reaction Monitoring) allows the quantitative determination of the concentrations of several metabolites of interest.

LC-MS systems do not have limitations on the size and volatility of the metabolites, which makes them more universal than GC-MS systems. However, the ionization with the EI method is not possible for LC-MS since the analyte enters the mass spectrometer in a huge excess of the liquid phase. Ionization methods in LC-MS have some limitations. The widely used electrospray ionization (ESI) works well for analytes that are charged in the LC mobile phase, but ESI cannot be used for the determination of non-charged analytes. Furthermore, the detection of a less-charged metabolite will be suppressed by a more-charged metabolite if both metabolites leave the LC column at the same time (i.e., they have the same retention time in LC protocol). Quantification of the concentrations of metabolites by the intensity of the MS signal using ESI requires special attention. Atmospheric pressure photo ionization and chemical ionization (APPI, APCI) also have some disadvantages. However, due to the wider range of analytes that can be analyzed by LC-MS compared to GC-MS and easier sample preparation (no chemical modification required), LC-MS is in many cases the preferred method for the metabolomic studies. Also, the advantage of ESI ionization is that it can be adjusted to be delicate enough to observe unfragmented analytes or less delicate to fragment analytes with the desired degradation depth.

An untargeted metabolomics study generally requires more powerful equipment. The ideal choice is MS with an FTICR mass analyzer coupled with chromatography or used in a shotgun approach (direct infusion of sample into the MS instrument without prior separation on a chromatographic column) [79]. LC-MS and GC-MS with TOF ion analyzer are less powerful but still useful instruments for untargeted metabolomics, especially if the identification of chemical metabolites is not the primary goal.

Tandem MS (MS/MS) instruments are very well suited for targeted metabolomics since they can highly selectively detect the presence of the metabolite of interest even in the presence of other metabolites that have not been separated chromatographically. The relatively affordable LC-MS/MS systems equipped with quadrupole mass analyzers can be used for the targeted metabolomic studies. GC-MS can also be used for targeted metabolomic if the metabolites of interest can be converted into volatile derivatives. The most affordable systems are GC-MS systems with a quadrupole analyzer.

For non-targeted metabolomics, the key factors are the high resolution of the method and a wide dynamic range for the determination of metabolites.

MS and especially MS/MS are sensitive methods and are powerful tools for the identification of metabolites. A single instrument can generate hundreds of thousands of MS/MS spectra per day, and the proper automated interpretation of the results is crucial. The straightforward approach for the automatic identification of a compound is the comparison with MS/MS spectra from spectral libraries, obtained using the reference samples. However, the libraries have spectra for just a small fraction of all possible compounds. High-resolution MS and computer-based approaches help to draw conclusions about the structure of the unknown compound. First, the analysis based on the mass differences caused by various content of stable isotopes in the same molecules helps to distinguish different compounds [80]. Second, the predicted fragmentation is calculated for virtually any possible small molecule since the fragmentation of molecules occurs by some rules, and the fragmentation products can be predicted. To infer the structure of unknown compound based on MS data, a predicted fragmentation tree (fragmentation of original molecule and fragmentation of the fragments) are used. The state-of-the-art tool, SIRIUS 4, uses a database of over 70 million unique chemical structures to reconstruct the combination of metabolites that generated a given pattern of fragmentation products [81].

### 6.3. Mass Spectrometry Imaging (MSI)

Currently, the isolation of organelles for metabolome analysis is the only way to reveal the intracellular distribution of the metabolites. Furthermore, some clues regarding the intracellular sites of metabolite formation can be obtained from information on the intracellular localization of metabolic enzymes. MSI provides a direct way to determine metabolites in a plant sample by the ionization of small spots on the surface of samples followed by MS analysis of the evaporated ions. In a recent study on 20-µm-thick cross-sections of a green asparagus spear, the spatial resolution by MSI reached was approximately 200 µm [82]. Light microscopy of stained sections allowed the identification of the type of tissue—ground, developing, epidermis (about 200 µm thick) and metaxylem and protoxylem in vascular bundles (with a diameter or about 400 µm). MSI with an FTICR mass spectrometer determined 11,365 mass features (MFs) in the sections with a spatial resolution of about 200 µm. Some MFs were recognized as specific metabolites. Thus, a health-promoting metabolite rutin (quercetin 3-*O*-rutinoside) was produced in the epidermis, developing stem tissue and protoxylem. All the MFs were divided into seven patterns using segmentation analysis. The distribution of the patterns across the section corresponded to the type of tissue.

MSIs have not yet reached a subcellular level of the analysis; however, it holds the promise to be a valuable tool in the fine determination of the location of the metabolites within tissues and cells.

### 6.4. Nuclear Magnetic Resonance (NMR) Spectroscopy

NMR is an alternative to MS methods for metabolomic studies. NMR instruments are much more expensive than MS systems besides the most advanced MS systems, such as MS with FTICR mass analyzer, which uses a magnetic field comparable in strength to the magnetic field used in NMR instruments. As reported in 2019, NMR is applied four to five times less often than MS [83]. NMR spectroscopy instruments measure chemical shifts in molecules containing atoms with a spin different from one, such as hydrogen (^1^H), stable isotopes of carbon (^13^C) and nitrogen (^15^N). An obvious advantage of NMR is the possibility of non-destructive measurements and measurements without sample preparation for both liquid, semi-liquid and solid samples. NMR measurements do not damage living samples; therefore, NMR provides a unique opportunity to measure metabolic fluxes in living objects. However, laborious sample preparation significantly improves the sensitivity and resolution.

NMR measurements are quantitative, highly reproducible, can be performed with high throughput, although longer measurements and measurements for both ^1^H and ^13^C strongly improve the results. The downsides of NMR are low sensitivity, especially for ^13^C and nitrogen ^15^N, and low resolving power. Thus, just up to the 100 most abundant metabolites can be recognized and quantified. However, in addition, NMR spectra have a vast number of minor peaks (more than ten thousand), which cannot be assigned for specific metabolites but can be utilized to characterize biological samples as molecular features (MF). Two-dimensional NMR (usually ^1^H and ^13^C) helps in the identification of some MFs as specific molecules; however, the ^13^C abundancy in natural samples is low and ^13^C NMR provides information on the metabolites with a high concentration in the sample. Thus, NMR is well-suited for untargeted metabolomic studies.

## 7. Metabolomics Studies

Metabolomics methods serve to determine chemical makeup at the level of small molecules in plant samples. Usually, plants of different varieties or grown in different conditions are compared. The information obtained using metabolomics helps in understanding the physiologic processes in plants and can be used on its for identification of metabolic markers of genotype performance or as diagnostic markers for the determination of specific stresses [84].

Methods of metabolomics, unlike genetic ones, require expensive equipment even for relatively simple tasks and greater care in sample preparation. It is not surprising that they are used much less frequently than genetic methods. However, metabolic studies are gradually becoming more common, with thousands of articles published annually. These studies can be divided into two main groups—purely metabolic studies and studies using other ohmic methods.

**Figure 2:**
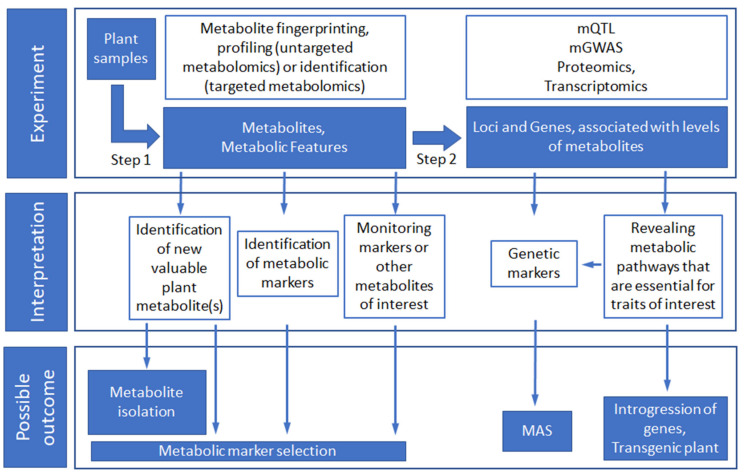
A general scheme of metabolic studies and their integration with QTL mapping and GWAS. Plant samples (leaves, roots, grains etc.) for metabolomics analysis should be collected with special care (see Section 6.1). Typically, plant samples are collected from different crop accessions, or the same accessions grown under different conditions, or samples are collected at different growth stages. In the first step, the metabolomics research methods are applied. The results of such studies are usually the determination of metabolites or metabolic features (see Section 6.1) with different concentrations between the samples. These metabolites can then be monitored as metabolic markers of agronomically important traits or as valuable traits by themselves. (see Section 7). Step 2 assumes integration of the genetic data. The metabolite concentrations obtained in the first step can be used as quantitative traits for QTL mapping analysis (which requires defined breeding protocol for generation of plant samples) or GWAS (which requires substantial number of genetically diverse plant samples of the cultivar of interest). It results in the identification of metabolic QTLs. Then, metabolic QTLs can be used for the development of genetic markers and their application for MAS (see Section 9). Also, valuable genes and gene sets can be identified in related and unrelated plant species to improve crops.

Pure metabolic studies can be focused on several goals. One of them is the identification of previously unknown secondary metabolites that are important by themselves, such as active substances of medicinal plants and essential flavor components of edible plants, coffee and tea. Another goal may be the identification of metabolic markers that report the current state of the plant, such as environment-dependent or stress responding primary and secondary metabolites or metabolites that can predict the desired traits. When the metabolites of interest are known or have been identified, they can be monitored in plants for the metabolic marker selection, plant diagnostics, testing of elicitors of secondary metabolites, etc. (Figure 2, Step 1).

Purely metabolomic research is not necessarily fast if the identification of metabolites is desired. An example of the decades-long work on the elucidation of the structures of active substances is the ginseng plant, which is widely used in traditional Chinese medicine. Medicinal as well as aromatic crops are high-value crops and are used in the cosmetic and pharmaceutical industries. Ginseng produces ginsenosides—secondary metabolites of the saponin family. Due to their complex steroid-like chemical structure, it takes a lot of effort to decipher this structure. At least 170 ginsenosides are currently known. Of them, over fifty ginsenosides were isolated from 2000 to 2019. The pharmacological effects of these metabolites still have to be determined [85]. Overall, plants are an important source of complex chemical substances. Natural products and semi-synthetic compounds account for about 30% of all FDA (the United States Food and Drug Administration agency) approved drugs [86].

## 8. Metabolic Markers and Their Performance

Since the metabolome is almost at the very end of the manifestation of the genetically encoded design of the organism, it has taken into account a large portion of gene–gene and gene–environment interactions. The metabolome contains “condensed information” [87] on the performance of the genotype in the specific growth conditions tested. Indeed, plant metabolites can be informative for the prediction of agronomically valuable traits. Metabolic markers are quantitative traits that are intensities of MS or NMR signals that represent their concentrations and are usually expressed in relative units. They can be correlated with quantitative phenotype traits. Here are some examples of the potential of metabolic markers for predicting agronomic traits.

A recent study demonstrated that corn yield and maturity biomass can be well predicted by metabolic parameters at a relatively early stage when the collar of the seventh leaf becomes visible (V7 stage), which occurs approximately a month after seeding, about a month before the corn begins to silk or about 3 months before the corn ripens. At this stage, the corn is growing rapidly; the kernel row determination is just beginning. The ratio of leaf starch and net carbon assimilation at the V7 stage correlated very well with the yield and maturity biomass (the correlation coefficients were -0.87 for yield and -0.96 correspondingly). The correlations were negative: the higher leaf starch content per net carbon assimilation, the lower the yield and maturity biomass of the three corn hybrids tested in the field [88].

An untargeted metabolomic study on the metabolome of rice seeds found that a number of metabolites are associated with the heading date of the plant that produced these seeds. Twenty-three metabolites of different classes (flavonoids, nucleic and amino acids and others) in mature seeds were associated with the heading date with the correlation coefficients +/− 0.50–0.68. Just to list some metabolites, they were flavonoid C-hexosyl-apigenin *O*-hexoside (R = 0.60), nucleic acid 5′-Deoxy-5′-(methylthio)adenosine (R = 0.59), anthocyanin Petunidin 3-*O*-rutinoside (R = 0.54) and amino acid L-Tryptophan (R = −0.53). Several metabolites with the highest correlation coefficients were not identified. It has been observed that the grain metabolome is highly dependent on the state of the grain—whether they are in the grain filling stage or mature seeds or germinating seeds. Among 800–850 metabolites observed, 372 metabolites were common for all three stages. Several metabolites were correlated with heading date at all three tested states of grains: fatty acid 9,10-epoxy-18-hydroxy-octadecanoic acid (R = 0.34 to 0.39), terpene Momilactone (R = 0.30 to 0.41) and a sugar derivative Methyl di-alpha-L-rhamnoside (R = 0.31 to 0.36) [89].

Fernandez et al. summarized coefficients of correlation of metabolic markers with phenotype traits from a number of various studies. Metabolites correlate with agronomic traits with |R| in the range of 0.2 to 0.8 [84].

The heritability of metabolite traits (metabolite levels) was investigated in a study of 2628 samples from 565 spring barley malting lines harvested in 3 years in two locations. Six phenotypic traits (filtering speed, wort clearness, extract yield, wort color, beta glucan and wort viscosity) and metabolomic data consisting of 24,018 MFs for corresponding wort samples were obtained. The MFs in this study were peaks in the NMR spectra that were not attributed to any particular metabolites. The heritability of almost 36% MFs was significantly different from 0, about 4% of MFs had moderate heritability (0.2 to 0.5) and 0.04% had high heritability (0.5 to 0.52). The NMR peaks with high heritability were similar to peaks of amino acids, such as tyrosine, phenylalanine and alanine. The MFs with the absence of heritability or low heritability perhaps were not the metabolite-related signals and appeared due to the extended range of chemical shifts obtained by NMR analysis. The correlation between MFs with significant heritability and phenotypic traits was weak, with |R| typically less than 0.30 [90].

## 9. Integration of Metabolomics Data with QTL and GWAS Data

A number of studies combine metabolomics data with genetic data and with other “omics” data. The most utilized approach is the genomic mapping of metabolites using metabolite-based (or metabolic or metabolome) QTL mapping (mQTL) and GWAS (mGWAS). As the quantitative traits, metabolite levels in QTL mapping and GWAS are treated as any other quantitative phenotypic trait. In QTL, traits mapping relies on recombination events in controlled crosses of two or more parents (biparental and multiparental populations). GWAS is performed using a set of diverse accessions of a crop of interest with an unknown degree of relationship [92]. In both approaches, differences in metabolite concentration between individuals are explained by the genetic control of the metabolite by the locus or loci. Mapping a metabolite to this specific locus (or loci) adds the genetic information known for this locus, such as a gene located in it. The opposite is also true—the associated metabolite may shed light on the possible function of gene(s) in the locus. From a practical point of view, mQTL and mGWAS allow to replace metabolite marker by a more convenient genetic marker for subsequent studies and breeding programs (Figure 2, Step 2).

In the metabolomic study on rice seeds at three states (seeds during grain filling, mature seeds and germinating seeds), mentioned in the previous chapter [89], mQTL application allowed the genetic determinants of the metabolic differences of two rice cultivars to be determined. The RILs (recombinant inbred lines) generated from the parent cultivars were substantially different in the metabolomic profiles of seeds, and more than 1500 mQTLs were found for each seed state that was associated with roughly 80% of all detected metabolites. The metabolites were controlled by one to seven mQTLs, and each mQTL mostly determined less than 20% of metabolite variation; however, above 1% of mQTLs determined more than 50% of metabolite variability. Thirty-five candidate genes were found within the determined mQTL. Overexpressing one of these genes in a transgenic plant confirmed its disturbing effect on the levels of amino acids [89].

Given the effectiveness of the mQTL and mGWAS methods for functional genomics, it is not surprising that they are actively used. More than 20 genome mapping studies of rice, wheat, corn, barley, tomato and rapeseed are listed in the review of Sharma et al. [90].

## 10. Metabolomics of Plant Stress Response 

Abiotic and biotic stresses cause global changes in the plant metabolome. The stress response is probably the area in which metabolomics has focused the most attention.

Plants respond to abiotic stress through phytohormone- and Ca^2+^-dependent signaling that activates transcription factors and induces the expression of stress-responsive genes. The stress also induces the generation of reactive oxygen species (ROS) [91].

The general scheme of response to pathogens is similar. The reception of biotic stress signals activates signaling pathways and hormonal regulation through primarily salicylic acid (mostly in response to biotrophic pathogens) and jasmonate with ethylene (mostly in response to necrotrophic pathogens), but other hormones (gibberellins, auxin, brassinolide, cytokinins and abscisic acid) also contribute to the response [92].

All these responses result in ROS generation and metabolic response.

### 10.1. Metabolomics for Abiotic Stress Responses and Tolerance

A metabolic response to drought stress was reported long before the term “metabolomics” was coined. The accumulation of amino acid proline was observed in 1954 in wilted rye. In the 1970–1990s, the list of metabolites accumulated in plants subjected to osmotic stresses (drought and soil salinity) was expanded [93]. The main role of elevated concentrations of these metabolites under osmotic stresses is to maintain turgor, and these metabolites have been named “compatible solutes” (not interfering with cell metabolism) and “osmolytes”. It has also been found that osmolytes, in addition to osmotic stresses, accumulate under a variety of other abiotic stresses.

To date, dozens of osmolytes are known. Roughly, they can be divided into three classes: soluble carbohydrates (sugars, raffinose family oligosaccharides and polyols or sugar alcohols), amino acids (including non-proteinogenic amino acids, such as 4-aminobutyrate) and polyamines and betaine with their derivatives, such as glycine betaine [12,94]. Transgenic sugar cane cultivars with increased synthesis of glycine betaine NXI-1T, NXI-4T and NXI-6T created by PT Perkebunan Nusantara XI (Persero) have an increased tolerance for water stress (see Section 5). It is assumed that under conditions of stress, osmolytes stabilize the lipid membrane structure, maintain proper protein folding and protect macromolecules and membranes from aggregation [94].

Aside from the common osmolytes stabilization effect on membranes and lipids, proline, perhaps the most common osmolyte, scavenges reactive oxygen species (ROS) singlet oxygen ^1^O_2_, an excited oxygen molecule with increased reactivity. This process occurs through a “physical” mechanism without the consumption of or damaging the proline. Proline also scavenges another common ROS superoxide anion O_2_^−^ in some chemical reaction(s) that converts proline to a product. Proline in both reactions is slower than ascorbate, but considering proline’s high concentration during stresses, its contribution to the total ROS-scavenging activity of metabolites becomes comparable to ascorbate [95].

Even a relatively small increase of proline is associated with improved crop performance. Thus, in ten tested common wheat cultivars, the level of proline in the leaves at heat stress during the grain filling stage increased by approximately three-fold, and this proline level at heat stress positively correlated with the yield [96].

Plant treatment by exogenous proline during various abiotic stresses at a concentration of 5–50 μM was protective for a number of plants. For example, the treatment of common wheat with 20 μM proline improved the grain yield [97].

Antioxidant defense is an important part of the stress response. By increasing the concentration of antioxidants, such as ascorbate, it is possible to increase the resistance of plants to stress [98].

The collection of osmolytes vary for different species [99]. Proline, sucrose, glucose are very common osmolytes for agricultural crops, and trehalose, a non-reducing disaccharide, is more common for non-vascular plants. Still, trehalose is considered an osmolyte in some studies of wheat and rice. Moreover, the metabolism of trehalose in vascular plants is interconnected with the abiotic stress response. Trehalose and its precursor, trehalose 6-phosphate (T6P), regulate stomatal function in guard cells. T6P and sucrose also reciprocally regulate their metabolism [99,100]. An improvement in abiotic stress tolerance following a reduction of T6P levels is a well-established observation, especially if the reduction is localized in developing reproductive organs [101]. The overexpression in developing maize ears of trehalose-6-phosphate phosphatase from rice reduced T6P concentration and improved yield by more than 10% in normal conditions and more than 30% in drought [102].

The comparison of changes in the metabolome of plants that are resistant and susceptible to a certain stress in response to this stress makes it possible to identify the metabolites responsible for the plant’s ability to adapt to this stress. These protective metabolites should be among the metabolites, the level of which increases more strongly in stress-resistant plants and vice versa, metabolites that hinder adaptation should be among those small molecules, the level of which in stress-resistant plants decreases more strongly.

In a recent comparison of the response to drought of a drought-resistant durum wheat cultivar Cappelli and drought-susceptible cultivar Colosseo, the increased accumulation of branch chain amino acids valine, leucine and isoleucine, as well other amino acids—proline, glycine, glutamate, alanine—and carboxylic acids acetate and malate in the seedlings of the resistant Cappelli was observed. In susceptible Colosseo, the changes were not so strong as in Colosseo, and glycine and glutamate were even slightly decreased in stress. Opposite to the amino acids, the level of sucrose in resistant Cappelli was reduced but raised in Colosseo. In these two contrasting cultivars, chloroplastic-like branched-chain amino acid amino-transferase TdBCAT from both A and B durum wheat genomes (TdBCAT-A and TdBCAT-B) were upregulated during drought stress only in the resistant Cappelli, while the expression level of TdBCAT-A virtually did not respond to the stress, and of TdBCAT-B responded by a small increase only. This observation raises the hypothesis that TdBCAT gene variants in Cappelli participate in the formation of drought stress tolerance [103].

A comparison of metabolic responses of lentil seedlings of drought-tolerant cultivar Elpida and drought-sensitive Flip03–24L showed that the drought stress strongly increased the level of glucose (20 fold) in both cultivars, while fructose and trehalose strongly decreased in the tolerant Elpida but strongly increased in sensitive Flip03-24L. Amino acids were raised in stress for both cultivars except tryptophan, which was decreased in sensitive Flip03-24L, and valine, which was decreased in Elpida and tended to be decreased in Flip03-24L. L-asparagine responded to stress in tolerant Elpida stronger than in susceptible Flip03-24L (20 fold vs. 7 fold). Carboxylic acid raised in stress with a stronger effect in the tolerant Elpida: seven–ten-fold in Elpida vs. five–six-fold in Flip03-24L for 2-ketoglutaric acid, five-fold vs. no change for malonic acid and four-fold vs. two–three-fold for citric acid [104].

A comparative metabolomic analysis of responses to cold in leaves of cold stress-resistant and susceptible cultivars of Tibetan hulless barley showed that the unique response of cold-resistant cultivar to cold stress was an increase in the levels of 6-methylmercaptopurine and coniferin [105].

It is generally accepted that environmental stress causes the increase of the levels of osmolytes, but the dynamics and magnitude of changes in each individual metabolite and the relationship between changes in the levels of different metabolites remains unclear.

### 10.2. Metabolomics for Biotic Stress Responses and Tolerance

Plants are constantly attacked by viruses, bacteria, fungi, nematodes, insects and animals. For their protection, plants use constitutively present metabolites, phytoanticipins that include saponins. Upon infection, plants induce oxidative stress and generate antimicrobial secondary metabolites of various classes named phytoalexins. Among phytoalexins are isoprenoids (terpenoids), alkaloids and flavonoids [106].

Pathogens induce changes in the concentrations of different classes of metabolites: amino acids, organic acids, fatty acids, phytohormones and polyamines [107].

To identify genes involved in response to a particular stress, a parallel analysis of the transcriptome is carried out. It allows correlations between the expression of certain genes and the appearance/accumulation of the metabolite of interest to be identified. The participation of correlating genes in the synthesis of metabolites is assumed based on the understanding of biosynthetic pathways. Thus, a recent metabolomic study revealed multiple changes in the metabolite level in response to pathogens in tomato [108]. The metabolomic response was different depending on the pathogen and elicitor type. Among other changes to the biotic stress-induced by fungus *Cladosporium fulvum*, the generation of falcarindiol was increased. Falcarindiol is an oxylipin and an acetylenic fatty acid. Based on the general understanding of falcarindiol biosynthesis, it was suggested that the key enzymes required for the generation of these acetylenic fatty acids are desaturases. To reveal the possible biosynthetic pathway that is activated and results in the generation of falcarindiol, the expression of genes was studied by RNA-seq in parallel with a metabolic study. Several types of pathogen/elicitors and several time points underwent parallel metabolome and transcriptome analysis. Among 40 putative desaturases found in the tomato transcriptome, mRNA expression of three desaturases were correlated best with falcarindiol accumulation in response to stress. Further, a putative decarbonylase was located in the same 20 kb locus, and its expression was also positively correlated with falcarindiol level. The generation of tomato lines with a knockout of these genes using CRISPR/Cas9 prevented the generation of falcarindiol, and genetic complementation tests using *Agrobacterium*-mediated transient overexpression of cDNA for these genes restored falcarindiol generation. Surprisingly, knockout tomato lines that were unable to generate falcarindiol were more resistant to pathogen compared to the falcarindiol-producing original tomato line. Even so, the combination of metabolomic study together with gene expression was able to uncover genes involved in specific metabolite biosynthesis.

## 11. Conclusions

Metabolomics is the latest and least mature among other major “omics”. It has made a significant contribution to understanding the chemical response of plants to stresses. However, the metabolomics studies on yield and yield components are sparse. More studies are required for more complete and consistent data. The ability to improve plant varieties in the long term requires the more active involvement of functional “omics”. It can be assumed that the metabolome, in comparison with transcriptome and proteome, is the closest to phenotype in terms of the degree of influence of gene–gene and gene–environment interactions. Therefore metabolites can be valuable markers of agronomically-important traits of agricultural crops. mQTL and mGWAS contribute greatly to the development of functional genomics. It can be expected that the methodology for the application of metabolomic methods in crop breeding will be intensively developed.

To date, a metabolism-based approach has proven useful for the creation of GM plants with improved traits, such as abiotic stress tolerance, better nutritional qualities etc. The introduction or suppression of one or many genes at once in one plant is an effective tool for obtaining transgenic plants with the desired characteristics. However, this reverse genetic approach is difficult to translate to breeding crops since there is no information of the performance of specific alleles and genes in the available genetic pools and wild relatives. There are two research gaps limiting the application of metabolomics: (1) the gap between experimentally obtained datasets of metabolite concentrations and their interpretation in terms of biochemical pathways and their perturbations and adjustments, and (2) a gap between the agronomically important traits and their underlying biochemical pathways. Thus, one of the future directions in metabolomics is the development of a framework that will allow to interpret a large set of metabolite concentration as readouts of specific metabolic processes. In the near future, the development and use of genetic markers obtained from mQTL mapping and mGWAS studies can be expected in crop breeding.

## Figures and Tables

**Table 1:** List of selected genetically modified (GM) crops that have been approved for public use. Accessions that were selected for the table are plants with genetically modified metabolic enzymes ^1^. The data and descriptions (with some modifications) are taken from "GM Approval Database" of International Service for the Acquisition of Agri-biotech Applications (ISAAA): Source: https://www.isaaa.org/gmapprovaldatabase/default.asp (accessed 30 September 2021).

GM Crop	GM Trait	Gene	Gene Source	Gene Product	Gene Function	Example(s) ^2^	Developer ^3^-Year of Approval ^4^
Tomato-*Lycopersicon esculentum*	Delayed fruit softening	pg (sense or antisense)	*Lycopersicon esculentum*	No functional polygalacturonase enzyme is produced (transcription of the endogenous enzyme is suppressed by a gene silencing mechanism)	Inhibits the production of polygalacturonase enzyme responsible for the breakdown of pectin molecules in the cell wall, and thus causes delayed softening of the fruit	SYN-ØØØØB-6	Z-1995
						FLAVR SAVR	M-1992
Melon-*Cucumis melo*	Delayed ripening/senescence	sam-k	*Escherichia coli bacteriophage T3*	S-adenosylmethionine hydrolase enzyme	Causes delayed ripening by reducing the S-adenosylmethionine (SAM), a substrate for ethylene production	35-1-N	A-1996
Tomato-*Lycopersicon esculentum*	Delayed ripening/senescence	anti-efe	*Lycopersicon esculentum*	Antisense RNA of 1-amino-cyclopropane -1-carboxylate oxidase (ACO) gene (no functional ACO enzyme is produced)	Causes delayed ripening by suppressing the production of ethylene via silencing of the ACO gene that encodes an ethylene-forming enzyme	Huafan No 1	HAU-1997
Tomato-*Lycopersicon esculentum*	Delayed ripening/senescence	accd	*Pseudomonas chlororaphis*	1-amino-cyclopropane-1-carboxylic acid deaminase enzyme	Metabolizes the precursor of the fruit ripening hormone ethylene, resulting in delayed fruit ripening	CGN-89322-3	M-1995
Carnation-*Dianthus caryophyllus*	Delayed ripening/senescence	acc (truncated)	*Dianthus caryophyllus*	Modified transcript of 1-amino-cyclopropane -1-carboxylic acid (ACC) synthase gene	Causes reduced synthesis of endogenous ethylene through a gene silencing mechanism and thus delayed senescence and longer vase life	FLO-ØØØ66-8	F-1995 (c.o.)
Pineapple-*Ananas comosus*	Delayed ripening/senescence	acc	*Ananas comosus*	1-aminocyclopropane-1-carboxylic acid synthase	Involved in catalyzing the penultimate step in ethylene biosynthesis	Rosé	DM-2016
	Modified fruit color	b-Lyc	*Ananas comosus*	Gamma-carotene	Increases lycopene accumulation using RNAi technology		
		e-Lyc	*Ananas comosus*	Delta-carotene	Increases lycopene accumulation using RNAi technology		
		Psy (Phytoene Synthase)	Tangerine *(Citrus reticulata)*	Phytoene	Increases lycopene and/or beta-carotene levels		
Sugarcane-*Saccharum* sp.	Drought stress tolerance	EcBetA	*Escherichia coli*	Choline dehydrogenase	Catalyzes the production of the osmoprotectant compound glycine betaine conferring tolerance to water stress	NXI-1T	P-2011
Sugarcane-*Saccharum* sp.	Drought stress tolerance	RmBetA	*Rhizobium meliloti*	Choline dehydrogenase	Catalyzes the production of the osmoprotectant compound glycine betaine conferring tolerance to water stress	NXI-4T	P-2013
Rice-*Oryza sativa* L.	Enhanced Provitamin A Content	crt1	*Pantoea ananatis*	Phytoene desaturase enzyme CRTI	Catalyzes the conversion of 15-cis-phytoene to all-trans-lycopene	Golden Rice	IRRI-2017
		psy1	*Zea mays*	Phytoene synthase ZmPSY1	Converts geranylgeranyl diphosphate into phytoene, and acts upstream of CRTI in the carotenoid biosynthesis pathway		
Cotton-*Gossypium hirsutum* L.	Low Gossypol	dCS	*Gossypium hirsutum* L.	dsRNA that suppresses the expression of d-cadinene synthase gene that encode d-cadinene synthase, a key enzyme involved in gossypol biosynthesis, thru RNAi pathway	Silence the endogenous dCS genes	TAM-66274-5	TAM-2018
Potato-*Solanum tuberosum* L.	Lowered Free Asparagine	asn1	*Solanum tuberosum*	Double stranded RNA	Designed to generate dsRNA to down regulate Asn1 transcripts which lowers asparagine formation	All 5 transgenes: Innate® Acclimate, Innate® Hibernate. All transgenes except VInv: Innate® Cultivate, Innate® Generate, Innate® Accelerate, Innate® Invigorate	JRS-2014 (2015 for Vlnv-containing accessions)
	Reduced Black Spot	ppo5 (polyphenol oxidase 5)	*Solanum verrucosum*	Double stranded RNA	Designed to generate dsRNA to down regulate Ppo5 transcripts which lowers black spot bruise development		
	Lowered Reducing Sugars	PhL	*Solanum tuberosum*	Double stranded RNA	Designed to generate dsRNA to down regulate PhL transcripts which lowers reducing sugars		
		R1	*Solanum tuberosum*	Double stranded RNA	Designed to generate dsRNA to down regulate R1 transcripts which lowers reducing sugars		
		Vlnv	*Solanum tuberosum*	Double stranded RNA	Downregulates VInv transcripts which lowers reducing sugars		
Maize-*Zea mays* L.	Male sterility	zm-aa1	*Zea mays*	Alpha amylase enzyme	Hydrolyses starch and makes pollen sterile when expressed in immature pollen	32138 SPT maintainer	DP-2011
Maize-*Zea mays* L.	Modified alpha amylase	amy797E	synthetic gene derived from *Thermococcales* spp.	Thermostable alpha-amylase enzyme	Enhances bioethanol production by increasing the thermostability of amylase used in degrading starch	Enogen™	Sy-2007
Maize-*Zea mays* L.	Modified amino acid	cordapA	*Corynebacterium glutamicum*	Dihydrodipicolinate synthase enzyme	Increases the production of amino acid lysine	Mavera™ Maize, Mavera™ YieldGard™ Maize	R-2003
Carnation-*Dianthus caryophyllus*	Modified flower color	dfr	*Petunia hybrida*	Dihydroflavonol-4-reductase (DFR) hydroxylase enzyme	Catalyzes the production of the blue-coloured anthocyanin pigment delphinidin and its derivatives	*All have dfr, some have bp40 or hfl:*Moondust™, Moonshadow™, Moonshade™, Moonlite™, Moonaqua™, Moonvista™	F-1995 or 1998 if with bp40 (c.o.)
		bp40 (f3′5′h)	*Viola wittrockiana*	Flavonoid 3′,5′-hydroxylase (F3′5′H) enzyme	Catalyzes the production of the blue-coloured anthocyanin pigment delphinidin and its derivatives		
		hfl (f3′5′h)	*Petunia hybrida*	Flavonoid 3′,5′-hydroxylase (F3′5′H) enzyme	Catalyzes the production of the blue-coloured anthocyanin pigment delphinidin and its derivatives		
		sfl (f3′5′h)	Sage *(Salvia splendens)*	Flavonoid 3′,5′-hydroxylase	Involved in the biosynthesis of a group of blue coloured anthocyanins called delphinidins	Moonique™ *(also has dfr, bp40 (f3′5′h))*	Su-2008 (c.o.)
		dfr-diaca	Carnation *(Dianthus caryophyllus)*	Dihydroflavonol-4-reductase enzyme	Functions in the biosynthesis pathway of the pink/ red-coloured anthocyandin 3-O-(6-O-malylglucoside) pigment in carnations	Moonpearl™, Moonberry™ *(also have dfr, bp40 (f3′5′h))*	
		cytb5	Petunia *(Petunia hybrida)*	Cytochrome b5	Cyt b5 protein acts as an electron donor to the Cyt P450 enzyme and is required for full activity of the Cyt P450 enzyme Flavinoid 3′ 5′ hydroxylase in vivo and the generation of purple/ blue flower colours.	Moonvelvet™ *(also has hfl (f3′5′h))*	Su-2008 (c.o.)
Rose-*Rosa hybrida*	Modified flower color	5AT	*Torenia* sp.	Anthocyanin 5-acyltransferase (5AT) enzyme	Alters the production of a type of anthocyanin called delphinidin	WKS82/130-4-1 *(also has bp40 (f3′5′h))*	Su-2008 (c.o.)
Argentine Canola-*Brassica napus*	Modified oil/fatty acid	te	*Umbellularia californica* (bay leaf)	12:0 ACP thioesterase enzyme	Increases the level of triacylglycerides containing esterified lauric acid (12:0)	Laurical™ Canola	M-1994
Argentine Canola-*Brassica napus*	Modified oil/fatty acid	Lackl-delta12D	*Lachancea kluyveri*	Delta-12-desaturase	Converts oleic acid to linoleic acid	DHA Canola	N-2018
		Micpu-delta-6D	*Micromonas pusilla*	Delta-6-desaturase	Convert a-linolenic acid to stearidonic acid		
		Pavsa-delta-4D	*Pavlova salina*	Delta-4-desaturase	Converts docosapentaenoic acid to docosahexaenoic acid		
		Pavsa-delta-5D	*Pavlova salina*	Delta-5-desaturase	Converts eicosatetraenoic acid to eicosapentaenoic acid		
		Picpa-omega-3D	*Pichia pastoris*	Delta-15-/omega-3-desaturase	Converts linoleic acid to a-linolenic acid		
		Pyrco-delta-5E	*Pyramimonas cordata*	Delta-5-elongase	Converts eicosapentaenoic acid to docosapentaenoic acid		
		Pyrco-delta-6E	*Pyramimonas cordata*	Delta-6-elongase	Convert stearidonic acid to eicosatetraenoic acid		
Argentine Canola-*Brassica napus*	Modified oil/fatty acid	OtD5E	*Ostreococcus tauri*	Delta-5 elongase	Catalyzes the decarboxylation Claisen-like condensation of two carbons from malonyl-CoA to C20:5n-3-CoA generating C22:5n-3-ß-keto-C oA, which is then converted to C22:5n-3-CoA by endogenous enzymes	LBFLFK	B-2019 (c.o.)
		OtD6D	*Ostreococcus tauri*	Delta-6 desaturase	Converts C18:2n-6 fatty acids into C18:3n-6 fatty acids		
		PiO3D	*Phytophthora infestans*	Omega-3 desaturase	Converts C20:4n-6 into C20:5n-3		
		PirO3D	*Pythium irregulare*	Two copies of the coding sequence for an omega-3 desaturase, cO3D(Pir)1 and cO3D(Pir)2	Converts C20:4n-6 into C20:5n-3		
		PlD4D	*Pavlova lutheri*	Delta-4 desaturase	Convert C22:5n-3 into C22:6n-3		
		PpD6E	*Physcomitrella patens*	Delta-6 elongase	Catalyzes the decarboxylation Claisen-like condensation of two carbons from malonyl-CoA to C18:3n-6-CoA generating C20:3n-6-ß-keto-CoA, which is then converted to C20:3n-6-CoA by endogenous enzymes		
		PsD12D	*Phytophthora sojae*	Delta-12 desaturase	Convert C18:1n-9 into C18:2n-6		
		TcD4D	*Thraustochytrium* sp.	Delta-4 desaturase	Converts C22:5n-3 into C22:6n-3		
		TcD5D	*Thraustochytrium* sp.	Two copies of the coding sequence for a delta-5 desaturase, cD5D(Tc)1 and cD5D(Tc)2			
		TpD6E	*Thalassiosira pseudonana*	Delta-6 elongase	Catalyzes the decarboxylation Claisen-like condensation of two carbons from malonyl-CoA to C18:3n-6-CoA generating C20:3n-6-ß-keto-CoA, which is then converted to C20:3n-6-CoA by endogenous enzymes		
Safflower-*Carthamus tinctorius* L.	Modified oil/fatty acid	fad2.2	*Carthamus tinctorius*	Fad2.2 gene-no functional enzyme is produced	Production of FAD2.2 (delta-12 desaturase enzyme) is suppressed by RNA interference	GOR-73226-6	G-2018
		fatB	*Carthamus tinctorius*	FatB gene-no functional enzyme produced	Production of FATB enzymes (acyl-acyl carrier protein thioesterases) is suppressed by RNA interference		
Soybean-*Glycine max* L.	Modified oil/fatty acid	gm-fad2-1 (partial sequence)	*Glycine max*	No functional enzyme is produced (expression of the endogenous fad2-1 gene encoding omega-6 desaturase enzyme was suppressed by the partial gm-fad2-1 gene fragment)	Blocks the formation of linoleic acid from oleic acid (by silencing the fad2-1 gene) and allows accumulation of oleic acid in the seed	Treus™, Plenish™	DP-2009
Soybean-*Glycine max* L.	Modified oil/fatty acid	gm-fad2-1 (silencing locus)	*Glycine max*	No functional enzyme is produced (production of endogenous delta-12 desaturase enzyme was suppressed by an additional copy of the gm-fad2-1 gene via a gene silencing mechanism)	Blocks the conversion of oleic acid to linoleic acid (by silencing the endogenous fad2-1 gene) and allows accumulation of monounsaturated oleic acid in the seed	DD-Ø26ØØ5-3	DP-1997
Soybean-*Glycine max* L.	Modified oil/fatty acid	fad2-1A (sense and antisense)	*Glycine max*	No functional enzyme is produced (production of delta-12 desaturase enzyme is suppressed by RNA interference)	Reduces desaturation of 18:1 oleic acid to 18:2 linoleic acid; increases the levels of monounsaturated oleic acid and decreases the levels of saturated linoleic acid in the seed	Vistive Gold™	M-2011
		fatb1-A (sense and antisense segments)	*Glycine max*	No functional enzyme is produced (production of FATB enzymes or acyl-acyl carrier protein thioesterases is suppressed by RNA interference)	Decreases the transport of saturated fatty acids out of the plastid, thereby increasing their availability to desaturation to 18:1 oleic acid; reduces the levels of saturated fatty acids and increases the levels of 18:1 oleic acid		
Soybean-*Glycine max* L.	Modified oil/fatty acid	Nc.Fad3	*Neurospora crassa*	Delta 15 desaturase protein	Desaturates certain endogenous fatty acids resulting in the production of stearidonic acid (SDA), an omega-3 fatty acid	MON87769	M-2011
		Pj.D6D	*Primula juliae*	Delta 6 desaturase protein	Desaturates certain endogenous fatty acids resulting in the production of stearidonic acid (SDA), an omega-3 fatty acid		
Potato-*Solanum tuberosum* L.	Modified starch/carbohydrate	gbss (antisense fragment)	*Solanum tuberosum*	No functional granule-bound starch synthase (GBSS) enzyme is produced; production of GBSS enzyme is suppressed by a gene silencing mechanism	Reduces the levels of amylose and increases the levels of amylopectin in starch granules	Amflora™, Starch Potato	B-2010
Tobacco-*Nicotiana tabacum* L.	Nicotine reduction	NtQPT1 (antisense)	*Nicotiana tabacum*	Antisense RNA of quinolinic acid phosphoribosyltransferase (QPTase) gene; no functional QPTase enzyme is produced	Suppresses the transcription of the QPTase gene, thereby reducing the production of nicotinic acid, a precursor for nicotine	Vector 21-41	V-2002 (c.o.)
Apple (*Malus x Domestica*)	Non-Browning	PGAS PPO suppression gene	*Malus domestica*	PGAS is a chimeric sense suppression transgene; it consists of 394 to 457 bp regions of four apple PPO (Polyphenol oxidase) genes (PPO2, GPO3, APO5, and pSR7) in tandem that upon transcription is designed to suppress the expression of these four members of the apple PPO gene family	Double stranded RNA (dsRNA)from the suppression transcript is processed into small interfering RNAs (siRNAs) that direct the cleavage of the target mRNA through sequence complementarity and suppresses PPO resulting in apples with a non-browning phenotype	Arctic™, Arctic™ Fuji Apple, Arctic™ "Golden Delicious" Apple	OSFI-2015
Argentine Canola-*Brassica napus*	Phytase production	phyA	*Aspergillus niger* var. van Tieghem	3-phytase enzyme	Increases the breakdown of plant phytates which bind phosphorus and makes the latter available to monogastric animals	Phytaseed™ Canola	B-1998
Maize-*Zea mays* L.	Phytase production	phyA2	*Aspergillus niger* strain 963	Phytase enzyme	Degrades phytate phosphorus in seeds into inorganic phosphate to be available to animals when used as feed	BVLA430101	OA-2009 (c.o.)

^1^ Enzymes conferring herbicide tolerance, nucleic acid-related enzymes such as barnase, DNA adenine methylase are not included; ^2^ Example(s) of the accessions: Trade Name, or Name, or Code; ^3^ Abbreviations for the companies/institutions in this table: A: Agritope Inc. (USA); B: BASF; DM: Del Monte Fresh Produce Company; DP: DuPont (Pioneer Hi-Bred International Inc.); F: Florigene Pty Ltd. (Australia); G: Go Resources Pty Ltd; HAU: Huazhong Agricultural University (China); IRRI: International Rice Research Institute; JRS: J.R. Simplot Co.; M: Monsanto Company (including fully and partly owned companies); N: Nuseed Pty Ltd; OA: Origin Agritech (China); OSFI: Okanagan Specialty Fruits Incorporated; P: PT Perkebunan Nusantara XI (Persero); R: Renessen LLC (Netherlands); Su: Suntory Limited (Japan); Sy: Syngenta; TAM: Texas A&M AgriLife Research University; V: Vector Tobacco Inc. (USA); **Z**: Zeneca Plant Science and Petoseed Company; ^4^ For an accession with several years of approval (for different countries and different applications-cultivation, feed or food) and for several accessions of the same type only the earliest year of approval is indicated. Since crop cultivation in most cases assumes some kind of utilization, accessions that have never received approval for food or feed are marked as “c.o.” (“cultivation only”) (not applicable for flowers, but still marked for the consistency).

## Data Availability

Not applicable.
